# Recurrent differentiated thyroid cancer: towards personalized treatment based on evaluation of tumor characteristics with PET (THYROPET Study): study protocol of a multicenter observational cohort study

**DOI:** 10.1186/1471-2407-14-405

**Published:** 2014-06-05

**Authors:** Jakob W Kist, Bart de Keizer, Marcel PM Stokkel, Otto S Hoekstra, Wouter V Vogel

**Affiliations:** 1 Department of Nuclear Medicine, The Netherlands Cancer Institute, Amsterdam, The Netherlands; 2 Department of Nuclear Medicine, University Medical Center Utrecht, Heidelberglaan 100, Utrecht 3584 CX, The Netherlands; 3 Department of Nuclear Medicine & PET research, VU University Medical Center, De Boelelaan 1117, Amsterdam 1081 HZ, The Netherlands

**Keywords:** Thyroid cancer, Recurrence, ^124^I, ^18^F-FDG, PET/CT, Cross-calibration, Thyropet

## Abstract

**Background:**

After initial treatment of differentiated thyroid carcinoma (DTC) patients are followed with thyroglobulin (Tg) measurements to detect recurrences. In case of elevated levels of Tg and negative neck ultrasonography, patients are treated 'blindly' with Iodine-131 (^131^I). However, in up to 50% of patients, the post-therapy scan reveals no ^131^I-targeting of tumor lesions. Such patients derive no benefit from the blind therapy but are exposed to its toxicity. Alternatively, iodine-124 (^124^I) Positron Emission Tomography/Computed Tomography (PET/CT) has become available to visualize DTC lesions and without toxicity. In addition to this, ^18^F-fluorodeoxyglucose (^18^F-FDG) PET/CT detects the recurrent DTC phenotype, which lost the capacity to accumulate iodine. Taken together, the combination of ^124^I and ^18^F-FDG PET/CT has potential to stratify patients for treatment with ^131^I.

**Methods/Design:**

In a multicenter prospective observational cohort study the hypothesis that the combination of ^124^I and ^18^F-FDG PET/CT can avoid futile ^131^I treatments in patients planned for ‘blind’ therapy with ^131^I, is tested.

One hundred patients planned for ^131^I undergo both ^124^I and ^18^F-FDG PET/CT after rhTSH stimulation. Independent of the outcome of the scans, all patients will subsequently receive, after thyroid hormone withdrawal, the ^131^I therapy. The post ^131^I therapeutic scintigraphy is compared with the outcome of the ^124^I and ^18^F-FDG PET/CT in order to evaluate the diagnostic value of the combined PET modalities.

This study primary aims to reduce the number of futile ^131^I therapies. Secondary aims are the nationwide introduction of ^124^I PET/CT by a quality assurance and quality control (QA/QC) program, to correlate imaging outcome with histopathological features, to compare ^124^I PET/CT after rhTSH and after withdrawal of thyroid hormone, and to compare ^124^I and ^131^I dosimetry.

**Discussion:**

This study aims to evaluate the potential value of the combination of ^124^I and ^18^F-FDG PET/CT in the prevention of futile ^131^I therapies in patients with biochemically suspected recurrence of DTC. To our best knowledge no studies addressed this in a prospective cohort of patients. This is of great clinical importance as a futile ^131^I is a costly treatment associated with morbidity and therefore should be restricted to those likely to benefit from this treatment.

**Trial registration:**

Clinicaltrials.gov identifier: NCT01641679

## Background

Differentiated thyroid cancer (DTC) is the most frequent endocrine tumor, with an annual incidence per 100.000 individuals of 1 – 3 in men and 2 – 4 in women [1]. In general, DTC has an excellent prognosis, and only 5 to 10% will die of their disease [2,3]. Prognosis is less favorable when the disease recurs after primary treatment. Local or regional recurrence occurs in 5 – 20% of patients [4]. Distant metastases develop in up to 10%, usually in the lungs and bones [5]. Recurrences are usually detected during the early years of follow-up, but may occur years later [6]. As in many diseases, early detection of recurrence improves outcome and survival, because limited disease load may allow surgical resection and/or effective treatment with radioactive iodine (^131^I). Follow-up is therefore necessary throughout the patients’ life. Therefore, even though DTC incidence is low, many patients are currently under surveillance for a possible recurrence (estimated 500.000 in the United States) [7,8].

The serum marker Thyroglobulin (Tg) plays a pivotal role in the follow-up of differentiated thyroid cancer. Serum Tg should be undetectable in DTC patients following effective thyroid remnant ablation with ^131^I, so that any detectable level reflects (neoplastic) thyroid tissue [9]. The level of serum Tg is related to the amount of neoplastic thyroid tissue; it has been estimated that 1 g of neoplastic thyroid tissue corresponds with a serum Tg of 1 ng/ml during thyroid hormone replacement therapy, and with 2 – 10 ng/ml following recombinant human thyroid hormone stimulating hormone (rhTSH) stimulation [10,11]. A serum Tg cut-off level ≥ 2 ng/ml following rhTSH is highly sensitive to identify patients in whom persistent tumor may be found with imaging techniques [12,13].

When recurrent DTC is suspected because of serum Tg above the cut-off level, several imaging tests may be performed to detect the exact sites of recurrence. The sodium/iodide symporter (NIS) mediates iodide uptake in the thyroid gland and thyroid cancer cells [14]. The ability of the thyroid to accumulate Iodide via NIS is the basis for scintigraphic thyroid imaging with radioiodine (using the gamma-emitting ^123^I) as well as for therapy using the beta-emitter ^131^I, which targets and destroys iodide-transporting benign and malignant thyroid cells. In thyroid cancer, the primary therapy is total thyroidectomy, which in practice is near-total to spare adjacent nerves and parathyroids. Postoperatively, ^131^I is used to ablate these postoperative thyroid remnants, and to detect (using post ^131^I whole body scintigraphy) and treat potential metastases [15-17]. With this approach, highly selective radiation doses can be achieved in tumor tissue, often much higher than with external radiotherapy.

Historically, the follow-up of patients with DTC included scintigraphy after a low activity of ^131^I if serum Tg was elevated, but this is no longer recommended because of poor sensitivity [18-23]. To date, whole body scintigraphy after ‘blind’ administration of high ‘therapeutic’ activity of ^131^I is performed in these patients, during withdrawal of thyroid hormone replacement to stimulate uptake of iodine in cells of thyroidal origin, both to diagnose and re-stage the potential recurrence and to initiate its treatment [18,24-28]. This strategy can be effective, but an estimated 38% - 50% of patients will have a negative post-therapeutic ^131^I whole body scan and/or no objective therapy effect [29,30]. Such patients will have received a total body irradiation of 450 millisievert (mSv) and may have suffered from side effects such as nausea, sialoadenitis, loss of taste, or reduced spermatogenesis. Furthermore, their risk of secondary malignancies has increased [31,32]. All induced by a treatment from which they derived no benefit. Also, the prolonged thyroid hormone withdrawal and subsequent hypothyroidism necessary for ^131^I therapy have major impact on quality of life, with a majority of patients suffering from significant changes in physical, psychological, and social well-being [33-37]. The high frequency of high activities ^131^I from which patients do not derive any benefit but are exposed to its toxicity and potential adverse oncological effects, has led to a search for new diagnostic tools to improve the selection of patients for such treatment.

Nowadays, ultrasound of the neck is applied to detect a local recurrence or regional lymph node metastases allowing direct biopsy to confirm the diagnosis. Nonetheless, ultrasound is limited to the neck only, and when negative in the presence of detectable Tg, whole body evaluation is required.

Recently, Iodine-124 (^124^I) has become available as a novel radionuclide for whole body Positron Emission Tomography/Computed Tomography (PET/CT) in the follow-up of DTC [38-41], with a promising diagnostic accuracy and a considerably lower radiation exposure than whole body scintigraphy after therapeutic activity of ^131^I [39]. Furthermore, recent experience has shown that ^124^I PET/CT images may be representative for the biodistribution and radiation dosimetry of subsequent therapy with ^131^I [42,43]. Thus, ^124^I PET/CT may allow for more accurate restaging of patients in a whole body procedure, perform dosimetry for subsequent ^131^I therapy and predict the outcome of the treatment. However, some recurrent DTC lesions do not accumulate iodine, which is correlated with tumor dedifferentiation and this implies a poor prognosis [5]. Patients suspected of non-iodine accumulating DTC, so far only evident after futile blind ^131^I therapy, require restaging before local or systemic therapy may be applied. Metabolic PET imaging with the glucose analogon ^18^F-fluorodeoxyglucose (^18^F-FDG), especially during (rh)TSH stimulation, has a high sensitivity to detect recurrent DTC in patients with detectable Tg and negative iodine scintigraphy [44]. It may correlate with a more aggressive tumor behavior and poor prognosis [45], and can help to select patients for other treatment modalities (surgery, external beam radiotherapy or multikinase inhibitors [46-48]). ^18^F-FDG PET/CT is currently applied only when prior treatment and imaging with therapeutic activity of ^131^I has proven to be ineffective [49]. The value of ^18^F-FDG PET/CT before ^131^I treatment has not been tested.

At the biological level, ^124^I and ^18^F-FDG uptake is related to expression of the sodium iodine symporter (NIS) [16], while ^18^F-FDG uptake is related to hexokinase-I (HKI) and Hypoxia-inducible factor 1-alpha (HIF-1α) activity [50,51]. The evaluation of the relation of ^124^I and ^18^F-FDG PET/CT imaging findings and histopathological parameters (such as thyroglobulin, TTF1, Ki-67 and Cytokeratine-19 staining) and response to ^131^I treatment will give more insight in the fundamental knowledge about DTC.

**Figure 1:**
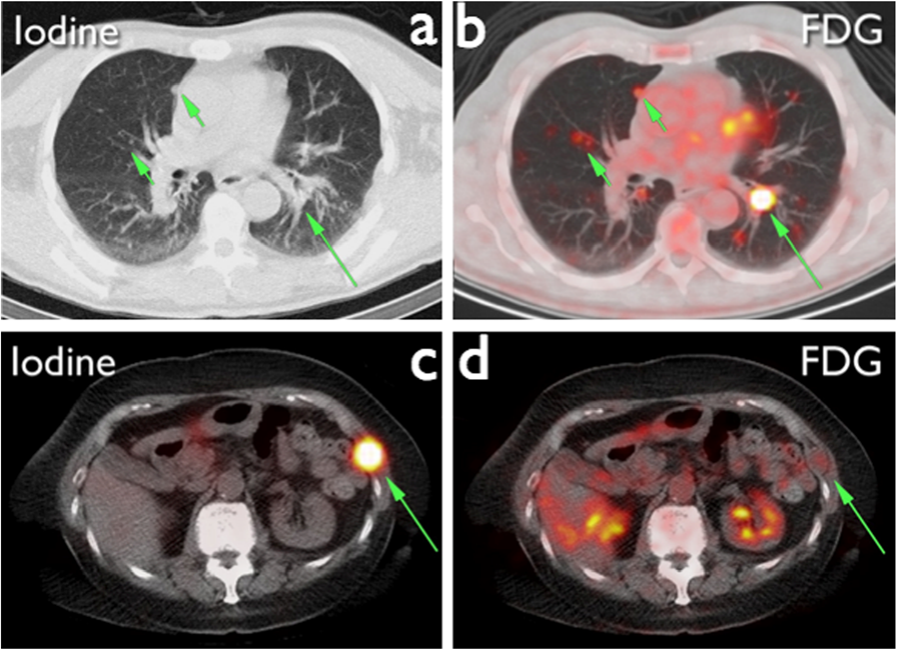
**Images from two different patients scanned with both **^**124**^**I and **^**18**^**F-FDG PET/CT.** The ^124^I PET/CT of patient 1 **(a)** shows multiple ^124^I negative pulmonary nodules, which are evidently ^18^F-FDG positive **(b)**. The thoracic wall lesion of patient 2 is clearly ^124^I avid **(c)** and showing no uptake on the ^18^F-FDG PET/CT **(d)**.

The present study aims to test the power of combined for detect and characterize DTC lesions in patients with suspected recurrence. Based on the characteristics of ^124^I and ^18^F-FDG PET/CT, it is reasonable to assume that a combined strategy of imaging and histopathological evaluation at the time of suspected recurrence will yield adequate information on the disease stage prior to treatment with ^131^I, regardless of tumor dedifferentiation, with a potential impact on clinical decision making. The combination of both entities has been suggested in proof of concept studies [52], illustrated in Figure 1, but needs proper testing, to increase fundamental knowledge about DTC and further improve treatment.

The multicenter design of this study requires highly standardized procedures for ^124^I PET/CT. Previously nationwide standardization was done for ^18^F-FDG PET/CT in the Netherlands, which eventually evolved into the European EARL accreditation system [53,54]. In order to compare the scans between centers calibration and standardization of the ^124^I PET/CT scans prior to the start of the study will be done a in quality assurance and quality control (QA/QC) program.

In summary, therapy with high activities of ^131^I for recurrent DTC is effective in many cases, but the current blind approach also leads to overtreatment, delay, and unnecessary decrease in quality of life in a significant number of cases. As we described, a combination of diagnostic tests has a potential to allow earlier and better restaging and selection for treatment. The proposed trial aims to test the value and optimal implementation of these new tests, standalone and in combination, to derive parameters for a new personalized strategy for diagnosis and treatment of patients with (suspected) recurrent DTC.

## Methods and design

### Study objectives

The primary aim of the study is to evaluate the value of combined imaging with ^124^I and ^18^F-FDG PET/CT in the prevention of futile treatment with high therapeutic activity of ^131^I. Interpretation of both PET-scans will lead to a positive or negative treatment proposal. This will be compared with the actual response on therapy. The definition of a futile treatment will be a negative post blind ^131^I therapy scintigraphy.

We define four secondary aims. Firstly, our aim is to organize a synchronized introduction and QA/QC of ^124^I PET/CT in the Netherlands. More specifically, we aimed to create a procedure for the cross-calibration of ^124^I PET/CT in a multicenter setting, which guarantees reliable and comparable quantification, and is practical to use. The procedure should result in calibration factors per scanner and an indication of a measurement threshold of the scanner, which is defined as the lowest activity that can be reliably quantified. The measurement threshold will be determined per vendor.

Secondly, translational correlation of ^124^I and ^18^F-FDG PET/CT with histopathology (where available) and treatment outcome will be done, in an explorative setting. The outcome of the treatment is defined as a positive or negative post-therapy scan. This scan and both ^124^I and ^18^F-FDG PET/CT will be correlated with histopathological features. The expression of different markers will be quantified in the samples. In this way we aim to determine which histopathological features of both primary tumor and metastatic lesions can predict outcome of the scans.

Thirdly, the study aims to investigate whether ^124^I PET/CT has the same diagnostic, dosimetric and prognostic yield during stimulation with rhTSH as with hormone withdrawal combined with low-iodine diet. Because ^124^I PET/CT will be performed both after stimulation with rhTSH and after withdrawal from levothyroxine it is possible to determine any differences in outcome from the two scan preparation strategies. Both visual assessment as the quantifiable data will be compared. As simultaneous administration of ^131^I and ^124^I is required this can only be done in selected.

Fourthly, we aim to compare ^124^I PET/CT and ^131^I scintigraphy dosimetry and correlate the results with clinical outcome. As ^124^I PET cannot be considered as the golden standard for dosimetry of iodine therapy the dosimetry based on ^124^I PET will be compared with ^131^I-scintigraphy dosimetry. An additional phantom study will be performed to correlate the results.

### Study design

This study is designed as a nationwide multicenter observational cohort study. The study population includes patients with biochemically suspicion (i.e. increase Tg levels) of recurrence of their previously completely removed thyroid carcinoma without evidence of local recurrence, planned for ‘blind’ therapeutic activity of ^131^I.

The patients to be included in the study should meet the following inclusion criteria:

1. Patients with a history of differentiated thyroid cancer

2. After complete thyroidectomy and ablation of functional remnants with ^131^I.

3. Planned for ‘blind’ treatment with high activity of ^131^I based on biochemically suspected recurrence, defined as a Tg-level above 2.0 ng/ml.

4. Ultrasonography of the neck performed < 2 months prior to inclusion.

If one of the following criteria is met patients will be excluded from the study:

1. Age < 18 years

2. Pregnancy

3. Incapacitated subjects

4. Contrast enhanced CT performed < 4 months prior to inclusion

5. ^131^I therapy performed < 12 months prior to inclusion

6. Indication for other therapy modality (i.e. surgery in case of a positive ultrasonography, radiotherapy, embolization or chemotherapy)

### Study endpoints

Primary endpoint is the number of futile high dose ^131^I treatments that could have been avoided by implementation of pre-therapy imaging based on post-therapy scintigraphy

Four secondary endpoints were defined: (1) Synchronized QA/QC of ^124^I PET in the Netherlands, (2) correlation of ^124^I PET/CT and ^18^F-FDG PET/CT with histopathological parameters, (3) correlation between ^124^I PET/CT findings during rhTSH and withdrawal combined with low-iodine diet and (4) correlation between ^124^I PET/CT and ^131^I-scintigraphy dosimetry.

### Study procedures

**Figure 2:**
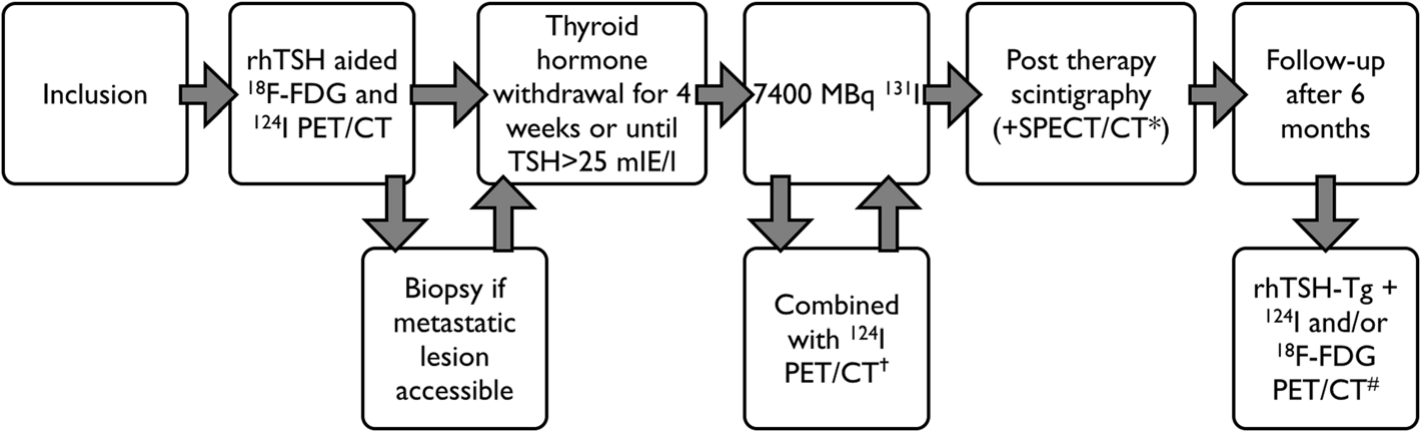
**Flow chart THYROPET study.** †Only in selected centers; if allowed according to local radiation safety regulations; *if available in center; ^# 124^I and ^18^F-FDG PET/CT only if pre-therapy scan was positive.

The study consists of four phases: pre-therapy, between pre-therapy and therapy, therapy and follow-up phase. For each phase the main study procedures are described below. Figure 2 shows an overview of the most important procedures.

#### Pre-therapy phase

Patients with biochemically confirmed recurrent DTC, will undergo ^18^F-FDG and ^124^I PET/CT imaging after pre-treatment with two injections of rhTSH. ^18^F-FDG will be administered and ^18^F-FDG PET/CT will be performed 60 minutes post injection. Subsequently, 74 megabecquerel (MBq) of ^124^I is administered intravenously. ^124^I PET/CT scans are then performed 24 and 96 hours after administration of ^124^I.

#### Between ‘pre-therapy phase’ and ‘therapy phase’

If either the ^18^F-FDG PET/CT or the ^124^I PET/CT shows metastatic lesions and it is possible to acquire a biopsy from the lesion, this will be done to correlate histopathological characteristics with both the result of the scans and the resection specimen of the original tumor. If multiple metastatic lesions are present on either of the scans, a biopsy will be pursued to acquire from every lesion, but only if the ^124^I or FDG uptake differs between the different lesions. This will be done in easily accessible metastatic lesions without large risks of complications and/or discomfort for the subject.

After the pre-therapy phase, subjects will start thyroid hormone withdrawal 4 weeks prior to ^131^I therapy. A low-iodine diet (LID) will be prescribed one week before the therapy.

#### Therapy phase

Subjects will undergo ^131^I therapy with 7400 MBq of ^131^I orally. In a subgroup of subjects (in selected centers) additional ^124^I PET/CT scans will be performed for dosimetric evaluation. Furthermore, the influence of the method of preparation for the scan, either withdrawal of thyroid hormone or rhTSH stimulation, will be evaluated. Seven days after administration of ^131^I a post-therapy scintigraphy is made, combined with SPECT/CT if available.

#### Follow-up phase

Six months after therapy both Tg and TSH levels will be determined after rhTSH administration. If the previous ^18^F-FDG PET/CT or the ^124^I PET/CT showed pathological uptake, that specific PET modality will be repeated. If both PET techniques were positive during the pre-therapy phase, both the ^18^F-FDG PET/CT and the ^124^I PET/CT will be repeated.

If another treatment modality, e.g. surgery, external beam radiotherapy or multikinase inhibitors, is indicated after the ^131^I therapy the data of this additional therapy will be collected as well. If a metastatic lesion is removed surgically the histopathological specimen will be collected for additional staining and reviewing by an expert endocrine pathologist.

### Additional protocol information

#### Histopathology thyroidectomy specimen

From every included subject original resection specimens of the thyroid will be collected and if possible additional staining will be done. All specimens will be reviewed and scored by an expert endocrine pathologist.

#### Histopathology biopsies

If one or more biopsies are acquired from the subjects between the pre-therapy and therapy phase they will be stored fresh-frozen and analyzed later.

#### Review panel

The local nuclear physician will assess all scans and, additionally, an expert review panel consisting of experienced nuclear physicians will assess every scan and every lesion individually as either positive or negative. Finally, the expert panel will discuss their disagreements to reach consensus on every scan and of every lesion in each scan.

### Sample size calculation

The power calculation is based on the (conservative) assumption that 40% of patients currently undergo a futile treatment. With approximately 50 evaluable patients per year in the Netherlands, we estimate we are able to include a minimum of 100 patients in 3 years. With a sample size of exactly 100 evaluable patients, a two-sided 95.0% confidence interval for a single proportion using the Pearson-Klopper method for constructing the confidence interval (exact binomial CI) will extend 10% from the observed proportion for an expected proportion of 40%.

### Recruitment and consent

The patients will be selected for potential participation by the endocrinologist. After consultation with on whether the patient is eligible the local principal investigator of the study the endocrinologist informs the patient. Informed consent is acquired at least a week later by the local principal investigator. See Additional file 1 for a list of participating centers.

### Withdrawal of individual subjects

Subjects of the study can leave the study at any time for any reason without any consequences. The investigator can decide to withdraw a subject from the study for urgent medical reasons. For every subject that decides to withdraw from the study a new subject will be included. In this way the number of subjects included will not be changed. If subjects withdraw from the study they will be offered regular follow-up.

### Follow-up of patients

Patients will receive standard follow-up according to the Dutch guidelines after the subject has completed the study.

### Premature termination of the study

The study relies on ^124^I PET/CT being predictive for ^131^I-treatment outcome. When 3 patients have been encountered with negative ^124^I PET/CT and positive post-therapy scintigraphy, the main clinical hypothesis can no longer be supported and the study will be stopped.

### Statistical analysis

Patient demographic data, tumor characteristics and data derived from the scans will be described in frequency tables. χ^2^-tests and trend tests (for ordered scales) will be used to determine whether a significant reduction in futile treatments could have been achieved by applying the ^124^I and ^18^F-FDG PET/CT. More in detail: interpretation of both PET-scans will lead to a positive or negative treatment proposal. This will be compared with the actual response on therapy. The definition of a futile treatment will be a negative post ‘blind’ ^131^I therapy scintigraphy. Additionally, accuracy measures such as sensitivity, specificity, positive and negative predictive value will be calculated from this data. Multivariate analysis will be performed whenever appropriate using logistic regression.

## Discussion

Since ^124^I has become available for PET scanning, the interest for its use in DTC has been high. More and more studies addressed its potential use in these patients. Furthermore, it is well known that during dedifferentiation of DTC, its tumor cells may become FDG avid and multiple studies have correlated ^18^F-FDG PET/CT with aggressiveness of DTC and the loss of iodine avidity. To our best knowledge no studies however addressed in a large prospective cohort of patients with recurrent thyroid cancer the additional value of these scan modalities in the prevention of futile ^131^I therapies. This is of great clinical importance as a futile ^131^I treatment is costly and not without short- and long-term side effects and should therefore be restricted to those who will likely to benefit from this treatment.

### Trial status

The Medical the Ethics Board of the Netherlands Cancer Institute approved the study for all participating centers. Subsequently, this approval has been checked by all participating centers. The study is recruiting patients Since December 2012. The estimated length of the study is four years.

## Abbreviations

123I/124I/131I: Iodine-123/Iodine-124/Iodine-131; DTC: Differentiated thyroid cancer; LID: Low-iodine diet; MBq: Megabecquerel; ml: Milliliter; mSv: Millisievert; ng: Nanogram; NIS: Sodium/iodide symporter; PET/CT: Positron emission tomography/Computed tomography; rhTSH: Recombinant human thyroid stimulating hormone; RT: Radiotherapy; Tg: Thyroglobulin; TSH: Thyroid stimulating hormone.

## Competing interests

This study is supported by an unrestricted grant by Cyclotron B.V. by providing the ^124^I free of charge.

## Authors’ contributions

JK is coordinating investigator Thyropet study and drafted the manuscript. OH, BdK, MS and WV participated in the design of the study, acquired funding for the study and critically revised the manuscript. All authors read and approved the final manuscript.

## Authors’ information

THYROPET study group. Dr. J.M.H. De Klerk, Department of Nuclear medicine, Meander Medical Center Amersfoort, The Netherlands. Dr. D. Huysmans, Catharina hospital Eindhoven, Department of Nuclear medicine, The Netherlands. Dr. H. van Tinteren, Department of epidemiology and statistics, Netherlands Cancer Institute – Antoni van Leeuwenhoek, The Netherlands. Dr. J.P. de Boer, The Netherlands Cancer Institute, Department of Medical oncology, The Netherlands. Prof. dr. J. Morreau, Department of Pathology, Leiden University Medical Center, The Netherlands. Drs. M. van der Vlies, Department of Nuclear medicine and PET research, VU University Medical Center, The Netherlands. Dr. M.C. Huisman, Department of Nuclear medicine and PET research, VU University Medical Center, The Netherlands. Dr. E.G.W.M. Lentjes, Department of Clinical Chemistry, University Medical Center Utrecht, The Netherlands. Prof. dr. J.W.A. Smit Department of Internal medicine, Radboudumc, The Netherlands. Dr. J. Lavalaye, Department of Nuclear medicine, St. Antonius hospital Nieuwegein, The Netherlands. Prof. dr. P.L. Jager, Department of Nuclear medicine, Isala clinics, The Netherlands. Dr. F. van der Zant, Department of Nuclear medicine, Medical Center Alkmaar, The Netherlands. Dr. C.J. Hoekstra, Department of Nuclear medicine, Medical Center Jeroen Bosch, The Netherlands. Prof. dr. M. Gotthardt, Department of Nuclear medicine, Radboudumc, The Netherlands. Drs. V.J.R. Schelfhout, Department of Nuclear medicine, Rijnstate hospital, The Netherlands. Dr. A.H. Brouwers, Department of Nuclear medicine, University medical Center Groningen, The Netherlands. Drs. A.B. van Dijk, Department of Nuclear medicine, Dr. Bernard Verbeeten Instituut, The Netherlands. Dr. W.I. de Bruin, Department of Nuclear medicine, Medisch Spectrum Twente, The Netherlands. Dr. I. Al Younis, Department of Nuclear medicine, Leiden University Medical Center, The Netherlands. Drs. F. Sivro, Department of Nuclear medicine, St. Lucas Andreas hospital, The Netherlands. Drs. J.A. Adam, Department of Nuclear medicine, Amsterdam Medical Center, The Netherlands. Dr. H.T.T. Phan, Department of Nuclear medicine, Medical Center Leeuwarden, The Netherlands. Dr. G.W. Sloof, Department of Nuclear medicine, Groene Hart Hospital, The Netherlands. Dr. N.R.L. Wagenaar, Department of Nuclear medicine, ZGT, The Netherlands. Dr. B.L.R. Kam, Department of Nuclear medicine, Erasmus MC, The Netherlands. Drs. M.R.J. ten Broek, Department of Nuclear medicine, Reinier de Graaf Groep, The Netherlands. Drs. F. Smit, Department of Nuclear medicine, Rijnland Ziekenhuis, The Netherlands.

## Pre-publication history

The pre-publication history for this paper can be accessed here:

http://www.biomedcentral.com/1471-2407/14/405/prepub

## Supplementary Material

Additional file 1 List of participating centers. Click here for file

## Research support

Dutch Cancer Society (NKI 2011-5024).

BV Cyclotron VU (Amsterdam, the Netherlands) provides I124 as an unrestricted study grant.
