# Supplementary material for: Recurrent differentiated thyroid cancer: towards personalized treatment based on evaluation of tumor characteristics with PET (THYROPET Study): study protocol of a multicenter observational cohort study
Source: BMC Cancer. 2014 Jun 5;14:405. doi: 10.1186/1471-2407-14-405 (PMC4058699; doi:10.1186/1471-2407-14-405)
Supplement: Additional file 1 — List of participating centers. [file 1471-2407-14-405-S1.pdf]

MS: 9461341321163148

Recurrent differentiated thyroid cancer: towards personalized treatment based on evaluation of tumour characteristics with PET (THYROPET Study): study protocol of a multicentre observational cohort study

List of participating centers

1. Amsterdam Medical Center
2. Antonius Ziekenhuis
3. Catherina ziekenhuis
4. Erasmus MC
5. Groene Hart Ziekenhuis
6. Instituut Vebeeten
7. Isala Klinieken
8. Jeroen Bosch Ziekenhuis
9. Leiden University Medical Center
10. Meander Medisch Centrum
11. Medisch Centrum Alkmaar
12. Medisch Centrum Leeuwarden
13. Medisch Spectrum Twente
14. Netherlands Cancer Institute
15. Radboudumc
16. Reinier de Graaf Groep
17. Rijnland ziekenhuis
18. Rijnstate ziekenhuis
19. Sint Lucas Andreas Ziekenhuis
20. Spaarne ziekenhuis
21. University Medical Center Groningen
22. University Medical Center Utrecht
23. VU Medical Center
24. ZGT Hengelo
